# Alteration of N-glycans and Expression of Their Related Glycogenes in the Epithelial-Mesenchymal Transition of HCV29 Bladder Epithelial Cells

**DOI:** 10.3390/molecules191220073

**Published:** 2014-12-01

**Authors:** Jia Guo, Xiang Li, Zengqi Tan, Wei Lu, Ganglong Yang, Feng Guan

**Affiliations:** 1 The Key Laboratory of Carbohydrate Chemistry & Biotechnology, Ministry of Education; School of Biotechnology, Jiangnan University, Wuxi 214122, China; E-Mails: petpan0107@gmail.com (J.G.); zengqtan@gmail.com (Z.T.); luyuweifeng@gmail.com (W.L.); glyanglife@jiangnan.edu.cn (G.Y.); 2 Wuxi Medical School, Jiangnan University, Wuxi 214122, China; E-Mail: xiangli@jiangnan.edu.cn

**Keywords:** epithelial-mesenchymal transition, glycogene, mass spectrometry, microarray, N-glycan

## Abstract

The epithelial-mesenchymal transition (EMT) is an essential step in the proliferation and metastasis of solid tumor cells, and glycosylation plays a crucial role in the EMT process. Certain aberrant glycans have been reported as biomarkers during bladder cancer progression, but global variation of N-glycans in this type of cancer has not been previously studied. We examined the profiles of N-glycan and glycogene expression in transforming growth factor-beta (TGFβ)-induced EMT using non-malignant bladder transitional epithelium HCV29 cells. These expression profiles were analyzed by mass spectrometry, lectin microarray analysis, and GlycoV4 oligonucleotide microarray analysis, and confirmed by lectin histochemistry and real-time RT-PCR. The expression of 5 N-glycan-related genes were notably altered in TGFβ-induced EMT. In particular, reduced expression of glycogene *man2a1*, which encodes α-mannosidase 2, contributed to the decreased proportions of bi-, tri- and tetra-antennary complex N-glycans, and increased expression of hybrid-type N-glycans. Decreased expression of *fuca1* gene, which encodes Type 1 α-L-fucosidase, contributed to increased expression of fucosylated N-glycans in TGFβ-induced EMT. Taken together, these findings clearly demonstrate the involvement of aberrant N-glycan synthesis in EMT in these cells. Integrated glycomic techniques as described here will facilitate discovery of glycan markers and development of novel diagnostic and therapeutic approaches to bladder cancer.

## 1. Introduction

Glycans play important roles in protein folding, cell-cell adhesion, host-pathogen interactions, and cell signaling. The study of glycans is inherently more complicated than that of nucleic acids and proteins, due to their more complicated structures [1]. Unlike the situation for nucleic acids and proteins, there is neither practical automatic synthesis nor high throughput sequencing techniques available for glycans, despite the great efforts and advances made in the synthetic/analytical chemistry of glycans. The sequencing of glycan structures has been focused on high resolution mass spectrometry (MS), which has produced an enormous amount of information on glycomics [2,3,4], however, the currently available structural information is often partial and detailed analysis requires significant instrumentation and expertise. Lectins, a class of proteins found in plants, bacteria, fungi, and animals that are known to bind specific oligosaccharide moieties, were used for the structural elucidation of mammalian glycans on glycoproteins [5]. The applications of lectins, including affinity enrichment, microarrays, immunohistochemistry, blotting, electrophoresis, and ELISA have been developed and used widely in the last decade [6,7,8,9,10].

Many studies have demonstrated that aberrant expression of glycosyltransferases is a common phenomenon in bladder cancer; e.g., expression of GnT-V and β,1-6-branched N-linked oligo-saccharides is related to low malignant potential [11,12]; ST3 β-galactoside α-2,3-sialyltransferase 1 (St3Gal1) plays a major role in the initial oncogenic transformation [13]. Glycan profiles, including composition and expression of glycoproteins, proteoglycans, and glycosphingolipids, show distinct differences between normal and cancer cells, and certain glycans are involved in signal transduction processes related to the cancer transformation and progression [14,15,16,17,18]. No studies to date have examined the global profile of N-glycans and related glycogene expression in bladder cancer.

The process of epithelial-to-mesenchymal transition (EMT) plays an important role in the invasion and metastasis of solid cancer cells. During EMT, cells acquire malignant properties and become more migratory and invasive [19,20,21]. Aberrant expression and functional roles of glycans and glycogenes in various types of cancer cells undergoing EMT have been documented in many studies; e.g.,: expression of β*3GalT4* gene and its product gangliotetraosylceramide (Gg4) is reduced in normal mouse mammary epithelial cells (NMuMG) [22]; *Mgat5* gene, which encodes GnT-V and generates β1,6-GlcNAc-branched N-glycans, is required for EMT [23]; N-acetylglucosaminyltransferase-III (GnT-III) affects TGFβ-induced EMT through prolongation of E-cadherin turnover and suppression of β-catenin/pSmad complex formation [24]; O-glycosylation of oncofetal fibronectin (onfFN) plays an important role during TGFβ-induced EMT in human prostate epithelial cell lines [25].

Here, we apply glycomic analysis to profile the global variation of N-glycans and their related genes during TGFβ-induced EMT in non-malignant bladder transitional epithelium HCV29 cells, using various high-throughput techniques. Altered expression of N-glycans was analyzed using lectin microarrays and MALDI-TOF-MS. Glycogene transcription levels were analyzed using glycogene microarrays. Integrated glycomic analysis revealed that several aberrant N-glycosylations are involved in TGFβ-induced EMT of HCV29 cells. Our findings are expected to facilitate the discovery of new glycan biomarkers for bladder cancer.

## 2. Results and Discussion

### 2.1. Establishment of a Model of TGFβ-Induced EMT

**Figure 1:**
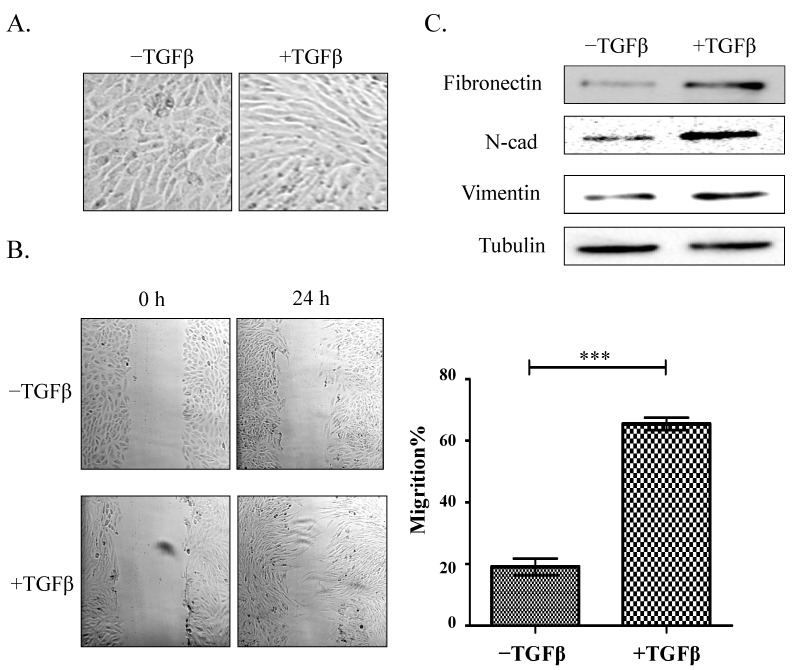
Effect of TGFβ treatment on HCV29 cell morphology, motility, and expression of EMT markers. (**A**) Morphological effects. Cells were cultured in 6-well plates and treated with 2 ng/mL TGFβ for 48 h. Photos were taken under phase-contrast microscopy; (**B**) Cell motility was assessed by wound assay. Image panels: cells were cultured in 24-well plates for 24 h and then treated with TGFβ for 48 h. Control and treated cells were scratched with a 200 μL pipette tip at the marked position. Cell were washed twice with fresh serum-free medium and then cultured in fresh culture medium without serum. Wounds were photographed at the marked position at 0 h and after 24 h incubation under phase-contrast microscopy. Right panel: migration ability analyzed by Scion Image program. ***, *p* ≤ 0.001; (**C**) Expression of EMT markers in control and TGFβ-treated cells. 10 μg protein/well were subjected to SDS-PAGE and western blotting, and expression of the EMT markers N-cadherin, vimentin, and fibronectin was analyzed by western blotting as described in the Experimental Setion. Tubulin was used as a control.

During EMT, cells undergo a morphological change and enhancement of motility [19,21]. HCV29 in normal medium had flattened epithelial morphology, while TGFβ-treated cells were converted to fibroblastic morphology (Figure 1A). Wound assays showed that motility was enhanced in TGFβ-treated cells relative to control cells, as expected (Figure 1B).

Loss of epithelial marker expression and increased expression of mesenchymal markers are the characteristic events of EMT [19,20,21]. We showed previously [22] that HCV29 cells express low levels of the mesenchymal markers N-cadherin, vimentin, and fibronectin and an undetectable level of the typical epithelial marker E-cadherin. In the present study, treatment of HCV29 cells with TGFβ resulted in significantly increased expression of N-cadherin, vimentin, and fibronectin relative to control cells (Figure 1C). Thus, TGFβ-treated HCV29 cells provide a useful model of TGFβ-induced EMT.

### 2.2. Glycosylation Analysis Using a Lectin Microarray during EMT in HCV29 Cells

Cell surface glycans are major components of cell membranes and play important roles in cell-cell and cell-extracellular matrix interactions. Aberrant glycosylation, catalyzed by glycosyltransferases and glycosidases, is often associated with malignant transformation and tumor progression [14,15,16,26]. In different bladder cancer cell lines, the N-glycosylation pattern of cadherin was related to the stage of invasiveness [27]; bi-, tri- and tetra-antennary structures were widely expressed in subunits of integrin α_3_β_1_, while high-mannose type structures were rare in HCV29 cells [28]; comparison the different glycosylation between HCV29 and bladder transitional cell carcinoma T24 treated with swainsonine (an inhibitor of Golgia-mannosidase II) showed that β1-6 branched tri- and tetraantennary complex-type glycans played an important role in the metastatic process [29]. However, no global glycan profiles have been studied in bladder cancer. Therefore, we combined lectin microarray analysis, MALDI-TOF-MS and glycogene microarray analysis, to elucidate the expression of glycans and glycogenes during TGFβ-induced EMT in HCV29 cells.

**Figure 2:**
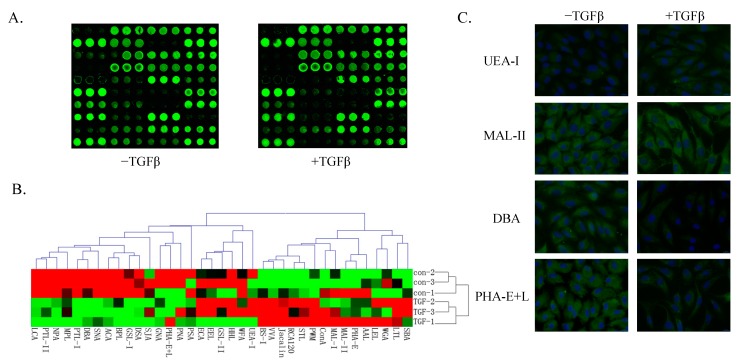
Variation of glycans recognized by lectin microarrays during TGFβ-induced EMT. (**A**) Fine structure of glycans was revealed by lectin microarray analysis as described in Materials and Methods. Fluorescence intensities from control samples (left) and TGFβ-treated samples (right) were scanned; (**B**) Variation of expression of glycans recognized by 37 lectins is presented as a “heatmap”. Red: fluorescence signal activation. Green: signal inhibition. Black: no clear link. Gray: missing data; (**C**) Altered expression of glycans evaluated by lectin histochemistry. Four lectins (Ulex europaeus agglutinin I [UEA-I], Maackia amurensis lectin II [MAL-II], Dolichos biflorus agglutinin [DBA], and Phaseolus vulgaris agglutinin E+L [PHA-E+L]) were applied, and lectin histochemistry was performed as described in Materials and Methods. Signals from a merge image of Cy3-conjugated lectins and DAPI staining of nuclei in control (left) and TGFβ-treated cells (right) are indicated (magnification 60×).

Lectin microarrays were performed for high-throughput analysis of glycan structures. Significant changes (>1.5-fold or <0.67-fold) of glycans were recognized by 15 different lectins during EMT (Table 1). Five glycan structures recognized by the lectins UEA-I, BS-I, MAL-II, RCA120 and PWM were up-regulated and 10 glycan structures recognized by the lectins PNA, GNA, SNA, NPA, PTL-II, DBA, PHA-E+L, DSA, PTL-I and GSL-I were down-regulated in the TGFβ-treated cells relative to control cells (Figure 2A; Table 1). Complete hierarchical clustering and visualization were performed using the Hierarchical Clustering Explorer 3.0 software program [30] (Figure 2B). Consistent with microarray findings, TGFβ-treated cells showed significantly increased binding signals with UEA-I and MAL-II and decreased binding signals with DBA and PHA-E+L by histochemical assays (Figure 2C).

**Table 1:** Variation of glycans determined by lectin microarray analysis of control *vs.* TGFβ-treated HCV29 cells.

Lectin	Fold Change	Specificity
Up-regulated		
UEA-I	2.78107	Fucα1-2Galβ1-4Glc(NAc)
BS-I	2.73611	α-Gal and α-GalNAc
MAL-II	1.88948	Sia2-3Galβ1-4Glc(NAc)
RCA120	1.63778	β-Gal
PWM	1.63695	GlcNAc
Down-regulated		
PNA	0.64744	Galβ1-3GalNAcα-Ser/Thr(T)
GNA	0.64599	Terminal α-1,3 mannose
SNA	0.62468	Sia2-6Galβ1-4Glc(NAc)
NPA	0.61967	Non-substituted α-1,6Man
PTL-II	0.32613	Gal
DBA	0.25435	αGalNAc,GalNAcα-Ser/Thr (Tn) and αGal
PHA-E + L	0.2388	Bisecting GlcNAc and biantennary N-glycans and tetra-antennary complex-type N-glycans
DSA	0.21933	GlcNAc
PTL-I	0.0513	αGalNAc and Gal
GSL-I	0.00542	αGalNAc,GalNAcα-Ser/Thr (Tn) and αGal

Lectin microarray analysis revealed a reduction of biantennary N-glycan structures and tetra-antennary complex-type N-glycans (recognized by PHA-E+L) in EMT. Similar changes in N-glycan structures have been reported in other cancers, e.g., and reduction of tri- and tetra-antennary N-glycan structures in hepatocellular carcinoma [31].

### 2.3. MALDI-TOF-MS Analysis of N-Glycan Profiles during EMT

**Figure 3:**
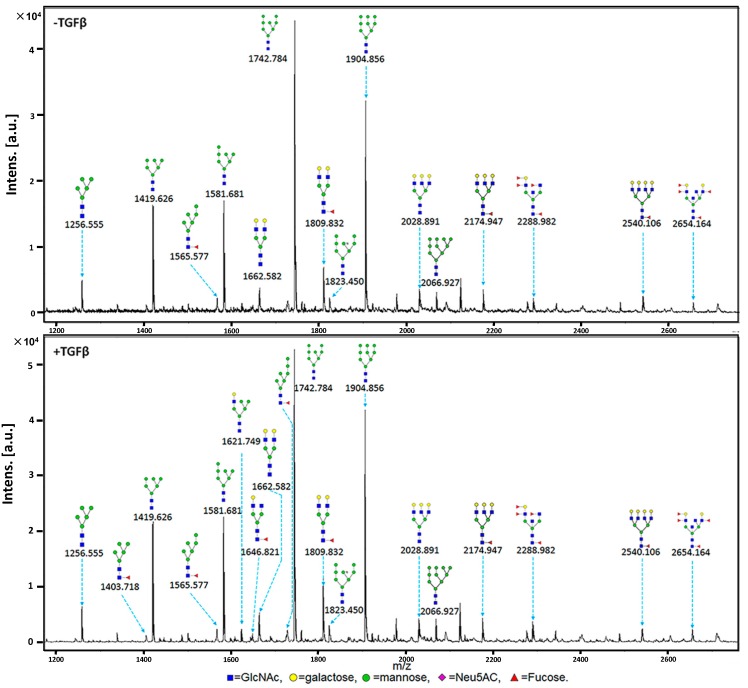
MALDI-TOF-MS spectra of N-glycans. HCV29 cells were cultured in a 10 cm dish (Corning; New York, NY, USA), and N-glycans from control and TGFβ-treated cells were separated and desalted as described in the Experimental. Lyophilized N-glycans were dissolved in methanol/H_2_O, and an aliquot of the mixture with DHB solution was spotted on an MTP AnchorChip sample target and air-dried. MALDI-TOF-MS was performed in positive-ion mode. Experiments were performed in triplicate, and representative N-glycan spectra are shown. Peaks (signal-to-noise ratio > 5) were selected for relative proportion analysis. Detailed structures were analyzed using GlycoWorkbench software. Proposed structures are indicated by *m/z* value.

N-glycosylation is involved in cell recognition, receptor activation, cell signaling, and cell adhesion in cancer [32]. Altered expression of N-glycans has been observed by mass spectrometry in many types of tumor cells. We applied MALDI-TOF/TOF-MS (tandem) to profile total N-glycans in TGFβ-treated cells as compared with control cells. Signal-to-noise ratios > 5 were the criterion for filtering distinct N-glycan peaks from the mass spectra, and N-glycans were annotated using GlycoWorkbench software [30]. Proposed N-glycan structures and their molecular weights are shown in Figure 3. 15 and 20 N-glycan structures were identified in control and TGFβ-treated cells, respectively. The 15 structures found in control cells were also present in TGFβ-treated cells, but with different intensities. Five structures were found only in TGFβ-treated cells (Table 2). Branching and linkage information for the N-glycan structures was obtained by MALDI-TOF/TOF-MS.

**Figure 4:**
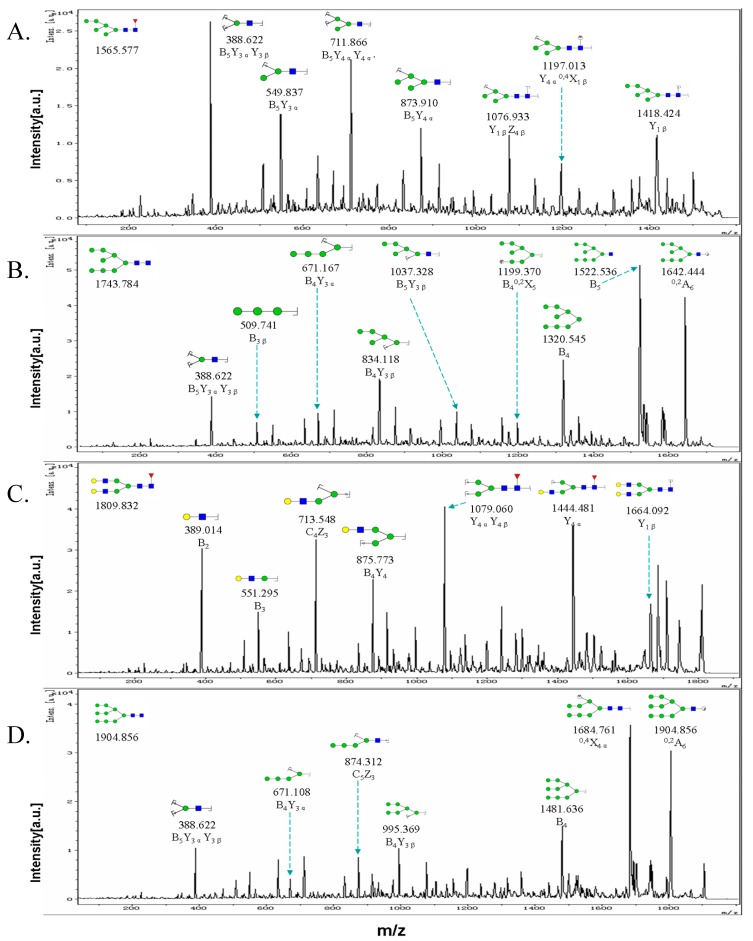
MALDI-TOF/TOF-MS/MS analysis of N-glycan precursor ions in MS spectra. Precursor ions were subjected to MS/MS analysis to obtain cleavages, including B, Y, C, and Z glycosidic cleavages and A and X cross-ring cleavages. Structures of cleavage ions and *m/z* values are shown in tandem mass spectra. Four major N-glycan peaks are indicated: *m/z* 1565.577 (**A**); 1743.784 (**B**); 1809.832 (**C**); and 1904.856 (**D**).

Two types of ions (B-,Y- and C-,Z-) were generated by glycosidic cleavages in tandem mass spectrometry, and cross-ring cleavages generated A-, X-type ions having greater energy. Tandem mass spectra of precursor ions with *m/z* 1565.577, 1743.784, 1809.832, and 1904.856 are shown in Figure 4. In the precursor ions with *m/z* 1743.784 and 1904.856, ion D (*m/z* 671.167) was identified as the most common fragment ion of high-mannose-type N-glycans, resulting from the loss of a 3-linked antenna and two reducing-end N-acetylglucosamine residues [33], e.g., B_4_Y_3α_. In the *m/z* 1809.832 ion, N-glycans were core fucosylated, as indicated by the fragment ions Y_4α_Y_4β_ (*m/z* 1079.060) and Y_1β_ (*m/z* 1664.092). Thus, MS in combination with MS/MS provided excellent insight into alteration of N-glycans during EMT.

Changes in the relative proportions of N-glycans detected in control and TGFβ-treated cells are summarized in Table 3. The proportion of complex-type N-glycan structures was lower in TGFβ-treated (40.0%) than in control cells (46.7%). In contrast, the proportions of high-mannose-type N-glycan (55.0% *vs.* 53.3%) and hybrid-type N-glycans (5.0% *vs.* 0.0%) were higher in TGFβ-treated than in control cells. The proportion of triantennary and tetra-antennary N-glycans were down-regulated in EMT. Fucosylation level was higher in EMT (50.0%) than in control cells (40.0%).

### 2.4. Glycogene Expression by Glycogene Microarray Analysis of TGFβ-Treated Cells

**Figure 5:**
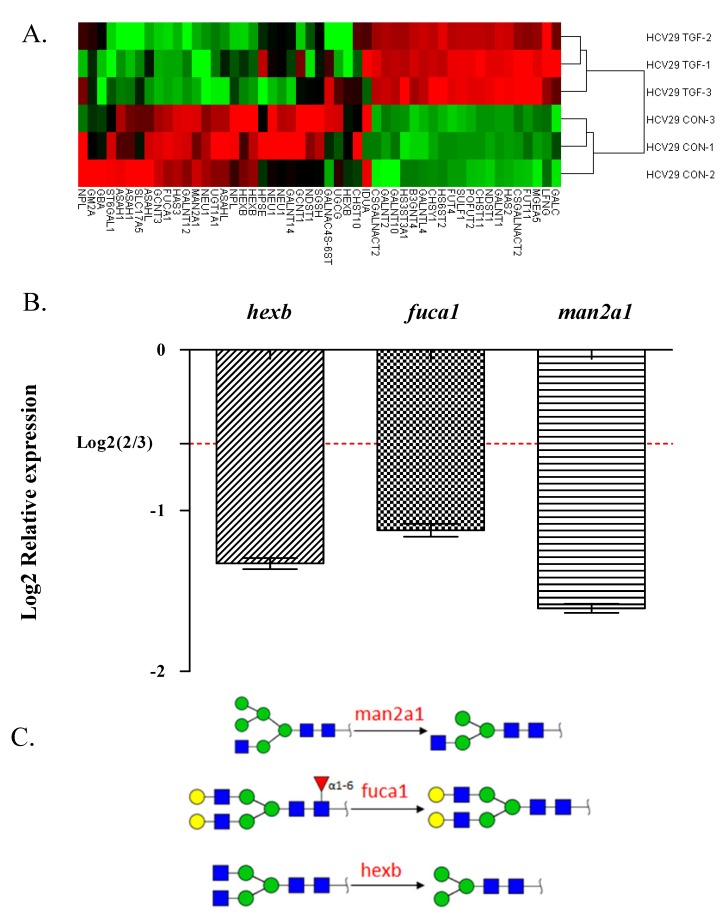
Gene expression during EMT revealed by glycogene microarray analysis. (**A**) Differentially expressed genes involved in the metabolism of glycoproteins, glycosphingolipids, and glycosaminoglycans in TGFβ-treated samples compared with control samples are shown as a “heatmap”. Red: genomic activation. Green: inhibition. Black: no clear link. Gray: missing data; (**B**) Gene expression of hexb, fuca1 and man2a1was analyzed by quantitative real-time RT-PCR as described in the Experimental. Experiments were performed in triplicate. Relative expression was analyzed using the 2^−ΔΔCt^ method and expressed as fold change relative to control cells. Expression of genes below Log_2_ (2/3) was significantly down-regulated. ***, *p* ≤ 0.001; (**C**) The relationship between glyco-molecules and their synthetic pathways with enzymes.

Among the 1260 human glycogenes covered by the GlycoV4 chip, a total of 178 were identified as differentially expressed following TGFβ treatment: 71 up-regulated genes and 107 down-regulated genes. To visualize the data, a “heatmap” was constructed using Cluster and Treeview software programs [30], and glycogenes from control and TGFβ-treated cells were clustered side-by-side in the dendrogram (Figure S1). Among the 178 differentially expressed genes, 51 were involved in glycan metabolism including 5 N-glycan-related genes (Table 4; Figure 5A), 42 were related to growth factors and their receptors, 18 were related to lectins and associated proteins, 10 were related to interleukins and associated receptors, and the remaining 57 were related to sulfotransferases, proteoglycans, glycoproteins, cytokines, adhesion molecules, or other molecules (Table S1).

**Table 2:** Proposed structures and their molecular ions in MALDI spectra of N-glycans from control and TGFβ-treated HCV29 cells.

NO.	Calculated (*m/z*)	Experimental (*m/z*)	Glycan Structure	Relative Intensity			
				Control		TGFβ-Treated	
				Average	CV(%)	Average	CV(%)
1	1079.377	1079.487	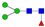	ND *	ND	1.50	0.47
2	1257.423	1256.555	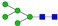	6.00	0.73	11.00	0.55
3	1403.481	1403.718	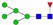	ND	ND	1.33	0.43
4	1419.476	1419.626	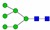	38.67	0.08	43.67	0.23
5	1565.533	1565.577	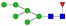	3.33	0.17	3.33	0.17
6	1581.528	1580.681	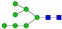	38.33	0.04	43.33	0.09
7	1622.555	1621.749	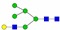	ND	ND	3.33	0.62
8	1647.587	1646.821	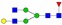	ND	ND	2.00	0
9	1663.581	1662.582	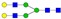	5.33	0.216	5.67	0.10
10	1727.586	1726.976	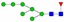	ND	ND	1.33	0.43
11	1743.581	1742.784	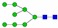	100.00	0	100.00	0
12	1809.639	1809.832	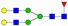	8.33	0.55	11.33	0.57
13	1823.547	1823.450	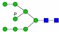	4.00	0	4.00	0.25
14	1905.634	1904.856	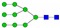	64.00	0.07	69.67	0.11
15	2028.713	2028.891	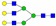	5.00	0.56	4.00	0.35
16	2067.687	2066.927	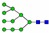	3.67	0.42	5.00	0.35
17	2174.772	2174.947	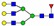	4.00	0.66	3.67	0.69
18	2287.819	2288.982	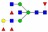	3.00	0	4.00	0.35
19	2539.904	2540.106	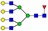	2.67	0.57	2.00	0.5
20	2652.951	2654.164	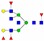	2.00	0	2.50	0.28

* ND: Not detected in the samples.

**Table 3:** Relative expression (proportions) of various types of N-glycans in control and TGFβ-treated HCV29 cells.

Glycan Type	Control	TGFβ-Treated Cells
High-mannose-type	53.3%	55.0%
Hybrid-type	0%	5.0%
Complex-type	46.7%	40.0%
Biantennary	20.0%	20.0%
Tri- and Tetra-antennary	26.7%	20.0%
Fucosylated	40.0%	50.0%

**Table 4:** Differential expression of N-glycan-related genes.

Gene Name	Genebank Acc.	Description	Fold Change
*st6gal1*	NM_173216	The encoded protein is a type II membrane protein that catalyzes the transfer of sialic acid from CMP-sialic acid to galactose-containing substrates.	0.61
*neu1*	NM_000434	The encoded protein is a lysosomal enzyme that cleaves terminal sialic acid residues from substrates such as glycoproteins and glycolipids.	0.54
*hexb*	NM_000521	Hexosaminidase B is the β-subunit of the lysosomal enzyme β-hexosaminidase that, together with the cofactor GM2 activator protein, catalyzes the degradation of ganglioside GM2 and other molecules containing terminal N-acetyl hexosamines.	0.54
*man2a1*	NM_002372	The encoded protein is a member of family 38 of the glycosyl hydrolases.	0.49
*fuca1*	NM_000147	The encoded protein is a lysosomal enzyme involved in the degradation of fucose-containing glycoproteins and glycolipids.	0.47

To confirm the above results, we detected expression of three N-glycan-related genes (*hexb*, *fuca1*, *man2a1*) by real-time RT-PCR. Both techniques showed greatly reduced expression of *hexb*, *fuca1*, and *man2a1* following TGFβ treatment (Figure 5B). These findings indicate that altered glycogene expression was involved in TGFβ-induced EMT in these cells. Increased glycan fucosylation was detected by UEA-I (which recognizes Fucα1-2Galβ1-4GlcNAc structure), corresponding to the down-regulated expression of *fuca1* in glycogene microarray analysis and real-time RT-PCR. Bi- and tetra-antennary complex-type N-glycan structures recognized by PHA-E+L were decreased in accord with *man2a1* expression (Figure 5C).

We compared N-glycan profiles of TGFβ-treated *vs.* control cells by MALDI-TOF/TOF-MS. Consistently with changes of *man2a1* transcription levels, TGFβ-treated cells showed a greatly increased proportion of hybrid glycan structures and a reduced proportion of complex-type structures. *man2a1* encodes α-mannosidase II, which hydrolyzes the terminal α-1,3 and -1,6 linked α-D-mannose residues of the high-mannose-type structure GlcNAcMan_5_GlcNAc_2_ [34,35,36,37]. In congenital dyserythropoietic anemia type II (HEMPAS; an autosomal recessive human genetic disorder), two glycoproteins termed bands 3 and 4.5 converted their biantennary complex-type oligosaccharides to hybrid-type structures with an M_5_ core, as a result of deficiency in either GlcNAc transferase II, β1,4-galactosyltransferase, or Golgi mannosidase II [38,39]. In Golgi mannosidase II-null mice, complex N-glycan structures on erythrocytes were reduced [40].

Our glycogene microarray analysis showed a clear reduction in transcription level of the *fuca1* gene. *fuca1* encodes type 1 α-l-fucosidase, which is involved in degradation of fucose-containing glycoproteins and glycolipids [41]. Fucosylation always occurs in the final step of glycoconjugate biosynthesis, and is related to malignant transformation, invasion, and metastasis [42,43]. Reduced expression of *fuca1* leads to fucosidase deficiency and enhanced fucosylation in cells. In the present study, MALDI-TOF-MS analysis of N-glycan profiles demonstrated increased fucosylation in TGFβ-treated cells, and lectin microarray analysis showed that the Fucα1-2Galβ1-4GlcNAc structure which was recognized by UEA-I, was almost 2-fold higher in TGFβ-treated than in control cells. These findings indicate that reduced *fuca1* expression contributes to increased fucosylation and that fucosylation plays an important role in TGFβ-induced EMT in bladder cancer.

## 3. Experimental Section

### 3.1. Cell Line and Culture

HCV29 cells were kindly donated by Dr. S. Hakomori (The Biomembrane Institute; Seattle, WA, USA). The cells were cultured in RPMI 1640 containing 10% fetal bovine serum (FBS; HyClone, Logan, UT, USA) and 1× penicillin/streptomycin (Gibco; Carlsbad, CA, USA) at 37 °C in 5% CO_2_ atmosphere.

### 3.2. Antibodies and Reagents

The antibodies used were mouse anti-N-cadherin IgG1 (Santa Cruz Biotechnology; Santa Cruz, CA, USA), rabbit anti-fibronectin IgG, mouse anti-vimentin IgG1, anti-gamma tubulin (Sigma-Aldrich; St. Louis, MO, USA), horseradish peroxidase (HRP)-labeled goat anti-mouse IgG, and HRP-labeled goat anti-rabbit IgG (Beyotime Institute of Biotechnology; Haimen, China). TGFβ was from BD Biosciences (San Jose, CA, USA). Other reagents were from Sigma unless described otherwise.

### 3.3. Total Protein Extraction and Western Blot Analysis

Cells were grown in regular medium for 24 h, the medium was then replaced by regular medium containing 2 ng/mL TGFβ, and cells were incubated for another 48 h. Cells were lysed with T-PER Tissue Protein Extraction Reagent (Thermo Scientific; San Jose, CA, USA). The solution was centrifuged at 12,000 rpm for 15 min. Protein concentration was determined using a BCA kit (Beyotime). Protein lysates were stored for western blot, lectin microarray, or N-glycan purification.

### 3.4. Wound Assay

Cells (2 × 10^4^/well) were cultured in 24-well plates overnight and treated with TGFβ. Three separate wounds were scratched in each well using a 200 μL pipette tip. Cells were rinsed twice with fresh serum-free medium and added with serum-free culture medium to eliminate cell proliferation. Wounds at the marked lines were photographed. After 24 h incubation, wounds at the marked lines were photographed again and the area occupied by moving cells was calculated [44].

### 3.5. Lectin Microarray Analysis and Data Analysis

Lectin microarray analysis and data analysis were performed as described previously [45,46]. In brief, lectin microarrays were produced by spotting 37 lectins from Vector Laboratories (Burlingame, CA, USA), Sigma-Aldrich, and Calbiochem Merck (Darmstadt, Germany) onto epoxysilane-coated slides. Glycoprotein samples labeled with the fluorescent dye Cy3 (GE Healthcare; Buckinghamshire, UK) were applied to the microarrays, which were then scanned with a GenePix 4000B confocal scanner (Axon Instruments; Union City, CA, USA). Normalized data for the experimental group and corresponding control group were compared to determine the relative change in protein glycosylation during EMT.

### 3.6. Lectin Histochemistry

Cells were cultured onto sterile coverslips in RPMI 1640 medium and treated with TGFβ for 48 h. The cells were then (i) fixed with 2% paraformaldehyde for 15 min and permeabilized with 0.2% Triton X-100 in PBS for 10 min; (ii) washed twice with PBS and blocked with 5% (w/v) BSA in PBS at 4 °C overnight; (iii) incubated with Cy3- labeled lectin (15–20 μg/mL) in 5% BSA for 3 h in the darkness and stained with DAPI (20 μg/mL) in PBS for 10 min; (iv) rinsed with PBS to remove extra dye; (v) mounted with Glycergel (DakoCytomation; Carpinteria, CA, USA) and observed using a fluorescence microscope (model Eclipse E600, Nikon; Tokyo, Japan).

### 3.7. Release and Purification of N-Glycans

Proteins were concentrated and desalted using a size-exclusion spin ultrafiltration unit (Amicon Ultra-0.5 10 KD device, Millipore; Billerica, MA, USA) [47] and denatured with 8 M urea, 10 mM DTT, and 10 mM IAM (Sigma-Aldrich, St. Louis, MO, USA). Denatured proteins were removed, and the samples in the ultrafiltration unit were further incubated with PNGase F (New England BioLabs; Ipswich, MA, USA) at 37 °C overnight to release N-glycans. N-glycans were eluted with Milli-Q water and desalted as described previously [47], dissolved in 1-butanol/methanol/H_2_O (5:1:1), and added into Sepharose 4B (Sigma-Aldrich) which was then equilibrated with methanol/H_2_O (1:1) and 1-butanol/methanol/H_2_O (5:1:1). After incubation for 45 min, N-glycans were washed with 1-butanol/methanol/H_2_O (5:1:1), eluted with methanol/H_2_O (1:1), and lyophilized.

### 3.8. MALDI-TOF/TOF-MS Analysis of N-Glycans

N-glycans were dissolved in 10 μL methanol/H_2_O (1:1), and spotted on an MTP AnchorChip sample target. Samples were air-dried, and 1 μL DHB solvent (20 mg/mL in methanol/H_2_O []) was added to recrystallize the glycans. N-glycans were analyzed by MALDI-TOF/TOF-MS (UltrafleXtreme, Bruker Daltonics; Bremen, Germany) in positive ion mode. *m/z* data were analyzed and annotated using GlycoWorkbench software.

### 3.9. Glycogene Microarray Analysis

RNA samples were prepared in the laboratory of Dr. S. Hakomori. GlycoV4 oligonucleotide chip analysis was performed by Gene Microarray Core E, Consortium for Functional Glycomics (CFG), Scripps Research Institute (La Jolla, CA, USA); results are available open-access at the CFG Functional Glycomics Gateway [48,49]. Differential expression of transcripts in TGFβ-treated *vs.* control cells was determined using cut-offs of fold change > 1.5, fold change < 0.67, and adjusted *p*-value < 0.05.

### 3.10. Real-Time RT-PCR

RNA was extracted using RNAPure Tissue Kit (CoWin Biotech; Beijing, China). The samples were treated with DNase I to rule out chromosomal DNA contamination. Each RNA sample (0.5 μg) was reversed transcribed using ReverTra Ace-α-^®^ (Toyobo; Shanghai, China). Real-time RT-PCR was performed with thermocycle conditions 95 °C for 10 min, 40 cycles of 95 °C for 10 s and 60 °C for 1 min in a 15 μL reaction system using UltraSYBR Mixture (CoWin Biotech). DNA products were analyzed using a CFX Manager (Bio-Rad; Berkeley, CA, USA). The primers used are listed in Table S2.

## 4. Conclusions

Aberrant glycans are always involved in cancer development and progression and are considered as potential cancer biomarkers. To identify glycan markers for bladder cancer, we applied a combination of different glycomic approaches during TGFβ-induced EMT in HCV29 cells. In comparison with control cells, TGFβ-treated cells showed: (i) reduced *man2a1* transcription, resulting in increased expression of hybrid-type glycans and reduced expression of complex-type glycan; and (ii) reduced *fuca1* transcription, resulting in increased appearance of various fucosylated N-glycans. Further studies using integrated glycomic techniques as described here will facilitate discovery of glycan markers and development of novel diagnostic and therapeutic approaches to bladder cancer.

## Supplementary Materials

Supplementary materials can be accessed at: http://www.mdpi.com/1420-3049/19/12/20073/s1.
