# Supplementary material for: Alteration of N-glycans and Expression of Their Related Glycogenes in the Epithelial-Mesenchymal Transition of HCV29 Bladder Epithelial Cells
Source: Molecules. 2014 Dec 1;19(12):20073–90. doi: 10.3390/molecules191220073 (PMC6271757; doi:10.3390/molecules191220073)
Supplement: Supplementary file 1 [file molecules-19-20073-s001.pdf]

Supplementary Materials

Figure S1. Differentially expressed genes (n = 178) during EMT as revealed by GlycoV4 chip analysis. Results are shown as a "heatmap". Red: genomic activation. Green: inhibition. Black: no clear link. Gray: missing data.

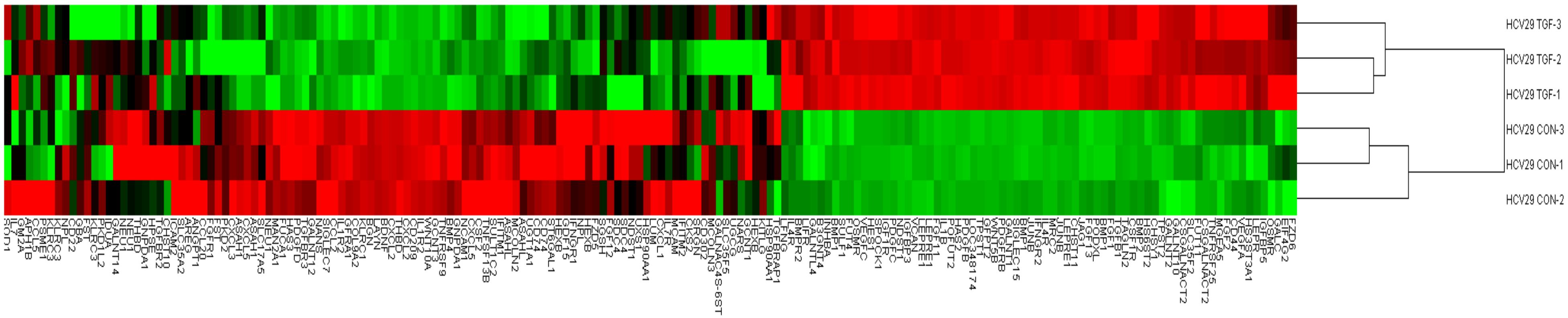

Table S1. Differentially expressed genes (n = 178) analyzed by GlycoV4 chips.

Gene Name	Genebank Acc.	Fold Change	Category
		Up-regulated	
LOC348174	NM_182619	34.89	CBP:C-type lectin
HAS2	NM_005328	11.38	Glycan-transferase
PODXL	NM_005397	8.32	Adhesion molecule
SIGLEC15	NM_213602	7.24	CBP:I-type lectin
BMP2	NM_001200	7.12	Growth factors and receptors
JAG1	NM_000214	5.43	Notch pathway
HES1	NM_005524	4.63	Notch pathway
TPST1	NM_003596	4.21	Sulfotransferase
GFPT2	NM_005110	3.91	Nucleotide sugar
PDGFB	NM_002608	3.89	Growth factors and receptors
JUNB	NM_002229	3.43	Miscellaneous
PDGFRB	NM_002609	3.22	Growth factors and receptors

Table S1. *Cont.*

Gene Name	Genebank Acc.	Fold Change	Category
TGFB1	NM_000660	3.13	Growth factors and receptors
JUNB	NM_002229	3.12	Miscellaneous
GALNT10	NM_017540	2.95	Glycan-transferase
WNT5B	NM_030775	2.80	Growth factors and receptors
IL1B	NM_000576	2.73	Interleukin and receptors
VCAN	NM_004385	2.71	CBP:C-type lectin
CHST11	NM_018413	2.51	Glycan-transferase
FGF1	NM_000800	2.48	Growth factors and receptors
SULF1	NM_015170	2.41	Glycan degradation
GALNTL4	NM_198516	2.37	Glycan-transferase
BMP1	NM_001199	2.36	Growth factors and receptors
INHBA	NM_002192	2.33	Growth factors and receptors
EIF4G2	uc001mjf	2.33	Miscellaneous
FZD6	uc003yli	2.17	Growth factors and receptors
FGF13	NM_004114	2.16	Growth factors and receptors
GALC	NM_001037525	2.05	Glycan degradation
CSF1R	NM_005211	1.97	Growth factors and receptors
BMP1	NM_006128	1.93	Growth factors and receptors
SPOCK1	NM_004598	1.92	Proteoglycan
HS6ST2	NM_147175	1.91	Glycan-transferase
OSMR	uc003jlm	1.90	Interleukin and receptors
IGF1R	NM_000875	1.89	Growth factors and receptors
LEPRE1	uc001chz	1.89	Proteoglycan
NDST1	uc003lsl	1.87	Glycan-transferase
VEGFA	NM_001033756	1.84	Growth factors and receptors
OSMR	NM_003999	1.84	Interleukin and receptors
BMP1	NM_006129	1.83	Growth factors and receptors

Table S1. *Cont.*

Gene Name	Genebank Acc.	Fold Change	Category
VEGFC	NM_005429	1.83	Growth factors and receptors
FUT4	NM_002033	1.82	Glycan-transferase
IFNGR2	NM_005534	1.81	Cytokine
FGF2	NM_002006	1.79	Growth factors and receptors
CHSY1	NM_014918	1.77	Glycan-transferase
GALNT1	NM_020474	1.74	Glycan-transferase
PDGFC	NM_016205	1.71	Growth factors and receptors
LEPR	NM_001003679	1.70	Growth factors and receptors
HS3ST3A1	NM_006042	1.69	Glycan-transferase
IGFBP5	NM_000599	1.66	Growth factors and receptors
IL4R	NM_000418	1.62	Interleukin and receptors
IL4R	NM_001008699	1.62	Interleukin and receptors
FGF11	NM_004112	1.61	Growth factors and receptors
CD44	NM_001001392	1.61	Proteoglycan
LFNG	NM_001040168	1.59	Glycan-transferase
CSGALNACT2	uc001jam	1.59	Glycan-transferase
MRC2	NM_006039	1.59	CBP:C-type lectin
SLC35F2	NM_017515	1.59	Nucleotide sugar
BMPR2	uc002uze	1.58	Growth factors and receptors
LIFR	NM_002310	1.58	Adhesion molecule
TPST2	NM_003595	1.55	Sulfotransferase
B3GNT4	NM_030765	1.53	Glycan-transferase
MGEA5	NM_012215	1.53	Glycan degradation
LEPRE1	NM_022356	1.53	Proteoglycan
GALNT2	NM_004481	1.53	Glycan-transferase
IGFBP3	NM_000598	1.52	Growth factors and receptors
LEPRE1	uc001chv	1.52	Proteoglycan

Table S1. *Cont.*

Gene Name	Genebank Acc.	Fold Change	Category
FUT11	uc001juz	1.51	Glycan-transferase
CSGALNACT2	NM_018590	1.51	Glycan-transferase
TNFRSF25	NM_148970	1.51	Miscellaneous
POFUT2	NM_015227	1.51	Glycan-transferase
TAGLN2	NM_003564	1.50	Miscellaneous
		Down-regulated	
HSP90AA1	NM_005348	0.67	Miscellaneous
APH1B	NM_031301	0.66	Notch pathway
KLRC3	NM_002261	0.66	CBP:C-type lectin
IL7	NM_000880	0.66	Interleukin and receptors
TGFBRAP1	NM_004257	0.66	Growth factors and receptors
GALNAC4S-6ST	NM_015892	0.66	Glycan-transferase
PSME1	NM_006263	0.65	Miscellaneous
SOD1	NM_000454	0.65	Miscellaneous
CD22	NM_001771	0.65	CBP:I-type lectin
IDUA	NM_000203	0.65	Glycan degradation
CHST10	NM_004854	0.65	Glycan-transferase
NARS	NM_004539	0.64	Human housekeeping
KLRC3	NM_007333	0.64	CBP:C-type lectin
CCL5	NM_002985	0.64	Chemokine
KLRC3	NM_002261	0.63	CBP:C-type lectin
GBA	NM_000157	0.63	Glycan degradation
SLC35F5	NM_025181	0.63	Nucleotide sugar
CD302	NM_014880	0.62	CBP:C-type lectin
HEXB	uc003kde	0.62	Glycan degradation
TGFBR2	NM_003242	0.62	Growth factors and receptors
PKD1L2	NM_001076780	0.62	CBP:C-type lectin

Table S1. *Cont.*

Gene Name	Genebank Acc.	Fold Change	Category
HPSE	NM_001098540	0.61	Glycan degradation
ST6GAL1	NM_173216	0.61	Glycan-transferase
GM2A	NM_000405	0.60	Glycan degradation
NEU1	NM_000434	0.60	Glycan degradation
NPL	uc001gpp	0.60	Glycan degradation
PKD2	NM_000297	0.60	CBP:C-type lectin
MCOLN3	NM_018298	0.60	Glycoproteins
SDC4	NM_002999	0.59	Proteoglycan
HSP90AA1	uc001ykx	0.59	Miscellaneous
NEU1	uc003sca	0.59	Glycan degradation
ANGPT1	NM_001146	0.59	Growth factors and receptors
FGF12	NM_004113	0.59	Growth factors and receptors
GCNT1	NM_001097634	0.59	Glycan-transferase
SGSH	NM_000199	0.58	Glycan degradation
NDST1	NM_001543	0.58	Glycan-transferase
ICAM2	NM_000873	0.57	CBP:I-type lectin
CKS2	NM_001827	0.57	Human housekeeping
SLC17A5	NM_012434	0.56	Glycan degradation
SLC35A2	NM_005660	0.56	Nucleotide sugar
FST	NM_006350	0.56	Growth factors and receptors
SDC4	NM_002999	0.56	Proteoglycan
KITLG	NM_003994	0.56	Miscellaneous
UXS1	NM_025076	0.55	Nucleotide sugar
ASAHL	NM_014435	0.55	Glycan degradation
UGCG	NM_003358	0.55	Glycan-transferase
GALNT14	NM_024572	0.55	Glycan-transferase
CXCL1	NM_001511	0.55	Chemokine

Table S1. *Cont.*

Gene Name	Genebank Acc.	Fold Change	Category
HEXB	NM_000521	0.54	Glycan degradation
HEXB	NM_000521	0.54	Glycan degradation
AREG	NM_001657	0.54	Growth factors and receptors
IFNGR1	NM_000416	0.54	Cytokine
SRGN	NM_002727	0.54	Proteoglycan
NEU1	NM_000434	0.54	Glycan degradation
IL7R	NM_002185	0.53	Interleukin and receptors
MCAM	NM_006500	0.53	Glycoproteins
IFITM2	NM_006435	0.53	Cytokine
ASAHL	uc003hjc	0.53	Glycan degradation
FGFR1	NM_023105	0.52	Growth factors and receptors
FZD5	NM_003468	0.51	Growth factors and receptors
GNPDA1	uc003lmh	0.51	Nucleotide sugar
GDF15	NM_004864	0.50	Growth factors and receptors
CCL20	NM_004591	0.50	Chemokine
NANS	NM_018946	0.49	Nucleotide sugar
MAN2A1	NM_002372	0.49	Glycan degradation
THBD	uc002wsu	0.49	Proteoglycan
BMP4	NM_130851	0.47	Growth factors and receptors
FST	NM_013409	0.47	Growth factors and receptors
FUCA1	NM_000147	0.47	Glycan degradation
LAYN	NM_178834	0.46	CBP:C-type lectin
CXCL3	NM_002090	0.46	Chemokine
NPL	NM_030769	0.45	Glycan degradation
LUM	NM_002345	0.43	Proteoglycan
NCAM1	NM_181351	0.42	CBP:I-type lectin
CD74	uc003lsf	0.42	Proteoglycan

Table S1. *Cont.*

Gene Name	Genebank Acc.	Fold Change	Category
UGT1A1	NM_000463	0.42	Glycan-transferase
ASAH1	NM_004315	0.41	Glycan degradation
ASAH1	NM_004315	0.41	Glycan degradation
CD74	NM_001025158	0.40	Proteoglycan
GNPDA1	NM_005471	0.39	Nucleotide sugar
CXCL5	NM_002994	0.39	Chemokine
IL1R2	uc002tbo	0.38	Interleukin and receptors
CXCL5	NM_002994	0.38	Chemokine
GALNT12	NM_024642	0.37	Glycan-transferase
BGN	NM_001711	0.36	Proteoglycan
TNFRSF9	NM_001561	0.36	Interleukin and receptors
TNFSF13B	NM_006573	0.34	Miscellaneous
PDGFD	NM_025208	0.33	Growth factors and receptors
CD209	NM_021155	0.32	CBP:C-type lectin
IL1R2	NM_004633	0.32	Interleukin and receptors
CSF2	NM_000758	0.30	Growth factors and receptors
SULT1C2	NM_001056	0.30	Sulfotransferase
MCOLN2	NM_153259	0.28	Glycoproteins
GCNT3	NM_004751	0.23	Glycan-transferase
THBD	NM_000361	0.21	CBP:C-type lectin
SIGLEC7	NM_016543	0.20	CBP:I-Type lectin
IFITM1	NM_003641	0.19	Cytokine
BDNF	NM_001709	0.19	Growth factors and receptors
COL9A2	NM_001852	0.17	Proteoglycan
TGFBR3	NM_003243	0.15	Growth factors and receptors
KLRC1	NM_002259	0.15	CBP:C-type lectin
WNT10A	NM_025216	0.15	Growth factors and receptors

Table S1. *Cont.*

Gene Name	Genebank Acc.	Fold Change	Category
GFRA1	NM_145793	0.11	Growth factors and receptors
CCL2	NM_002982	0.10	Chemokine
CXCL2	NM_002089	0.09	Growth factors and receptors
CXCL2	NM_002089	0.08	Growth factors and receptors
HAS3	NM_005329	0.08	Glycan-transferase

Table S2. Primers used in this study.

Primers	DNA Sequence (5'-3')	Use
hHexb-S	GTTGATGACCAGTCTTTCCCA	Real-time PCR for *hexb*
hHexb-AS	TCACCATACGGACATCATTTG	
hFuca1-S	TATCGTCGTGACATGGCATT	Real-time PCR for *fuca1*
hFuca1-AS	CCTTTCTTGGAAGATGGGAAC	
hMan2a1-S	ACCAACGAGATTCGGTCTTG	Real-time PCR for *man2a1*
hMan2a1-AS	CCTGGAGATGGATTTGAAACA	
